# More about the doubling degeneracy operators associated with Majorana fermions and Yang-Baxter equation

**DOI:** 10.1038/srep08102

**Published:** 2015-01-29

**Authors:** Li-Wei Yu, Mo-Lin Ge

**Affiliations:** 1 Theoretical Physics Division, Chern Institute of Mathematics, Nankai University, Tianjin 300071, China

## Abstract

A new realization of doubling degeneracy based on emergent Majorana operator Γ presented by Lee-Wilczek has been made. The Hamiltonian can be obtained through the new type of solution of Yang-Baxter equation, i.e. 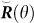 -matrix. For 2-body interaction, 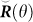 gives the “superconducting” chain that is the same as 1D Kitaev chain model. The 3-body Hamiltonian commuting with Γ is derived by 3-body 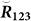 -matrix, we thus show that the essence of the doubling degeneracy is due to 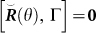. We also show that the extended Γ′-operator is an invariant of braid group *B_N_* for odd *N*. Moreover, with the extended Γ′-operator, we construct the high dimensional matrix representation of solution to Yang-Baxter equation and find its application in constructing 2*N*-qubit Greenberger-Horne-Zeilinger state for odd *N*.

The Majorana mode^1^,^2^,^3^,^4^ has attracted increasing attention in physics due to its potential applications in topological quantum information processing^5^,^6^,^7^. Specifically, the degenerate ground state in Majorana mode serves as topologically protected states which can be used for topological quantum memory.

In the Ref. 8, Lee and Wilczek presented a new operator Γ that provided the doubling degeneracy for the Hamiltonian formed by Majorana fermions to overcome the conceptional incompletion of the algebraic set for the Majorana model. Following the Ref. 8, the Majorana operators *γ_i_*'s satisfy Clifford algebraic relations:
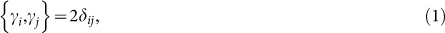
and the Hamiltonian takes the form
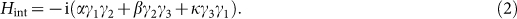
The algebra in equation (1) is conceptually incomplete. Besides the parity, the nonlinear operator Γ is introduced^8^
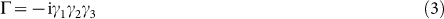
to form the set
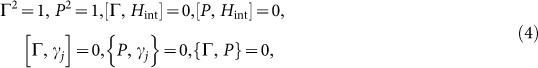
where *P* implements the electron number parity, and *P*^2^ = 1. The emergent Majorana operator Γ and parity operator *P* lead to the doubling degeneracy at any energy level, not only for the ground state.

On the other hand, based on the obtained new type of solution 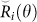 of Yang-Baxter equation (YBE), which is related to Majorana operators, the corresponding Hamiltonian can be found by following the standard way^9^, i.e. the Hamiltonian 
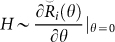
. We find that the Hamiltonian derived from 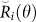 is 1D Kitaev model^1^. Moreover, because 1 + 1D 3-body S-matrix can be decomposed into three 2-body S-matrices based on YBE, we construct the 3-body Hamiltonian from 3-body S-matrix and find its doubling degeneracy. Hence, the advantage of parametrizing the braiding operator *B_i_* to 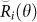 is that the desired Hamiltonian associated with Majorana operators can be derived from 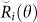.

Now let us first give a brief introduction to the Majorana representation of braiding operator as well as the solution of Yang-Baxter equation.

The non-Abelian statistics^10^ of Majorana fermion (MF) has been proposed in both 1D quantum wires network^7^ and 2D *p* + i*p* superconductor^2^. For 2*N* Majorana fermions, the braiding operators of Majorana fermions form braid group *B*_2*N*_ generated by elementary interchanges 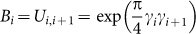 of neighbouring particles 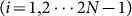 with the following braid relations:
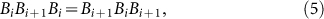

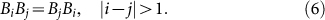


The Yang-Baxter equation (YBE)^9^,^11^,^12^ is a natural generalization of braiding relation with the parametrized form:
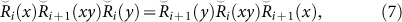
where *x*, *y* stand for spectral parameters,
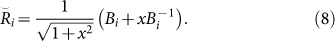


The solutions of equation (7) was intensively studied by Yang, Baxter, Faddeev and other authors^11^,^12^,^13^,^14^,^15^,^16^,^17^,^18^,^19^,^20^ in dealing with many body problems, statistical models, low-dimensional quantum field theory, spin chain models and so on. We call this type of solutions type-I.

Based on Ref. 21 there appears a new type of solutions called type-II^22^,^23^,^24^,^25^. By introducing a new variable *θ* as 
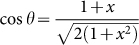
 and 
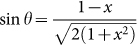
, we have
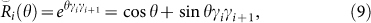
then the YBE reads^26^:
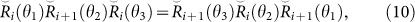
with the constraint for three parameters *θ*_1_, *θ*_2_ and *θ*_3_:
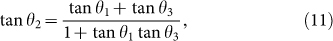
i.e. the Lorentzian additivity by 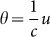. It is well known that the physical meaning of *θ* is to describe entangling degree, which is |sin 2*θ*| for 2-qubit^23^. The type-II solution of YBE 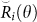 means the operation between two Majorana fermions, *γ_i_* and *γ_I_*_ + 1_. Because *γ_i_*'s satisfy Clifford algebraic relations:
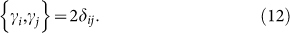
Then the solution 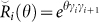 transforms the Majorana fermions *γ_i_* and *γ_i_*_ + 1_ in the following way:
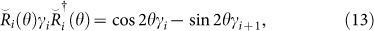

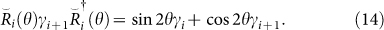


Since the solution of Yang-Baxter equation can be expressed in Majorana form, the following problems arise: (i) How to understand the Γ-operator intuitively on the basis of the concrete MF model generated by YBE; (ii) How to obtain the 3-body Hamiltonian, which possesses the doubling degeneracy, from YBE; (iii) What is the relationship between Γ-operator (as well as extended Γ′) and the solution 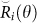 of YBE.

In this paper, we show that the emergent Majorana operator Γ is a new symmetry of 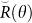 as well as Yang-Baxter equation. Due to the symmetry, the 3-body Hamiltonian derived from YBE holds Majorana doubling. We also present a new realization of doubling degeneracy for Majorana mode. Moreover, we discuss the topological phase in the “superconducting” chain. The generation of Greenberger-Horne-Zeilinger (GHZ) state via the approach of YBE is also discussed.

## Results

### Topological phase in the derived “superconducting” chain

The topological phase transition in the derived “superconducting” chain based on YBE is discussed. We find that our chain model is exactly the same as 1D Kitaev model. Let us first give a brief introduction to 1D Kitaev model.

1D Kitaev's toy model is one of the simplest but the most representative model for Majorana mode^1^,^4^. The model is a quantum wire with N sites lying on the surface of three dimensional *p*-wave superconductor, and each site is either empty or occupied by an electron with a fixed spin direction. Then the Hamiltonian is expressed as the following form:
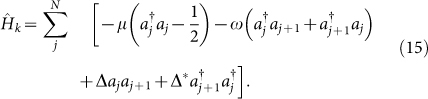
Here 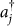, *a_j_* represent spinless ordinary fermion, *ω* is hopping amplitude, *μ* is chemical potential, and Δ = |Δ|*e*^−*iφ*^ is induced superconducting gap. Define Majorana fermion operators:
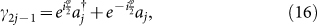

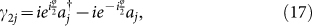
which satisfy the relations:
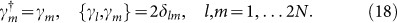
Then the Hamiltonian is transformed into the Majorana form:
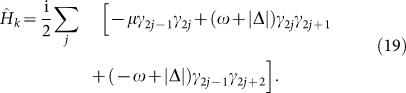


An interesting case is *μ* = 0, *ω* = |Δ|. In this case, the Hamiltonian turns into Majorana mode corresponding to topological phase:
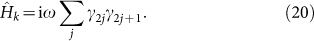
The above Hamiltonian has two degenerate ground states, |0〉 and |1〉 = *d*^†^|0〉. Here *d*^†^ = *e*^−i*φ*/2^(*γ*_1_ − i*γ*_2*N*_)/2 is a non-local ordinary fermion. The degenerate states can be used for topological quantum memory qubits that are immune to local errors.

Now let us construct the “superconducting” chain based on the solution 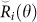 of YBE. We imagine that a unitary evolution is governed by 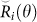. If only *θ* in unitary operator 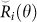 is time-dependent, we can express a state |*ψ*(*t*)〉 as 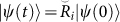. Taking the Schrödinger equation 
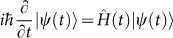
 into account, one obtains:
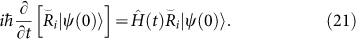
Then the Hamiltonian 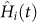 related to the unitary operator 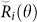 is obtained:
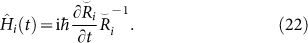
Substituting 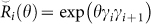 into equation (22), we have:
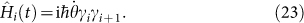
This Hamiltonian describes the interaction between *i*-th and (*i* + 1)-th sites with the parameter 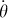. Indeed, when 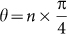, the unitary evolution corresponds to the braiding progress of two nearest Majorana fermion sites in the system, here n is an integer and signifies the times of braiding operation.

If we only consider the nearest-neighbour interactions between MFs and extend equation (23) to an inhomogeneous chain with 2N sites, the derived “superconducting” chain model is expressed as:
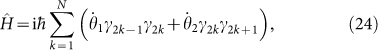
with 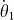 and 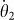 describing odd-even and even-odd pairs, respectively.

**Figure 1:**
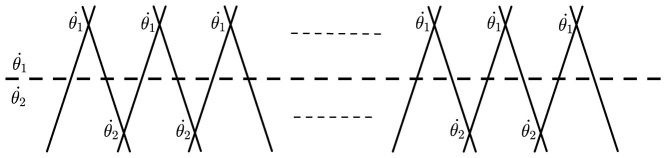
The nearest neighbouring interactions of 2N Majorana sites described by the “superconducting” chain. Each solid line represents a Majorana site, and the crossing means the interaction. The dashed line divides the interactions into two parts that are described by 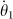 and 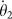 respectively. When 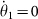, 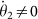, the first line and the last line are free, and the Hamiltonian corresponds to topological phase.

Now we give a brief discussion about the above chain model in two cases (see Fig. 1):

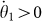
, 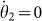.In this case, the Hamiltonian is:
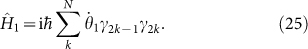
As defined in equation (16) and (17), the Majorana operators *γ*_2*k* − 1_ and *γ*_2*k*_ come from the same ordinary fermion site k, 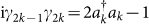 (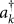 and *a_k_* are spinless ordinary fermion operators). 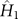 simply means the total occupancy of ordinary fermions in the chain and has U(1) symmetry, *a_j_* → *e^iϕ^a_j_*. Specifically, when 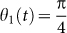, the unitary evolution 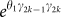 corresponds to the braiding operation of two Majorana sites from the same k-th ordinary fermion site. The ground state represents the ordinary fermion occupation number 0. In comparison to 1D Kitaev model, this Hamiltonian corresponds to the trivial case of Kitaev's. In Fig. 1, this Hamiltonian is described by the intersecting lines above the dashed line, where the intersecting lines correspond to interactions. The unitary evolution of the system 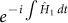 stands for the exchange process of odd-even Majorana sites.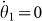, 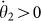.In this case, the Hamiltonian is:
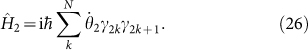
This Hamiltonian corresponds to the topological phase of 1D Kitaev model and has 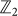 symmetry, *a_j_* → −*a_j_*. Here the operators *γ*_1_ and *γ*_2*N*_ are absent in 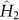, which is illustrated by the crossing under the dashed line in Fig. 1. The Hamiltonian has two degenerate ground state, |0〉 and |1〉 = *d*^†^|0〉, *d*^†^ = *e*^−*iφ*/2^(*γ*_1_ − *iγ*_2*N*_)/2. This mode is the so-called Majorana mode in 1D Kitaev chain model. When 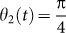, the unitary evolution 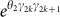 corresponds to the braiding operation of two Majorana sites *γ*_2*k*_ and *γ*_2*k* + 1_ from *k*-th and (*k* + 1)-th ordinary fermion sites, respectively.

Thus we conclude that our Hamiltonian derived from 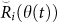 corresponding to the braiding of nearest Majorana fermion sites is exactly the same as the 1D wire proposed by Kitaev, and 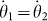 corresponds to the phase transition point in the “superconducting” chain. By choosing different time-dependent parameter *θ*_1_ and *θ*_2_, we find that the Hamiltonian 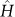 corresponds to different phases.

### New realization of Majorana Doubling based on Γ-operator

The important progress had been made to establish the complete algebra for the Majorana doubling by introducing the emergent Majorana operator Γ^8^:
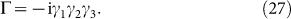
In Ref. 8, the concreted realization of the operators was presented in terms of Pauli matrices. On the other hand, as pointed out in Ref. 27, there is the transformation between the natural basis and Bell basis for
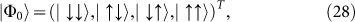

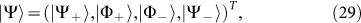
where
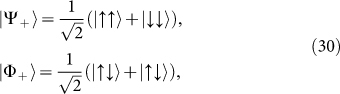

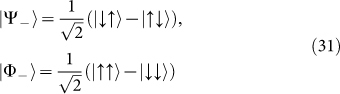
through the matrix *B_II_*:
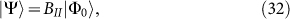
where
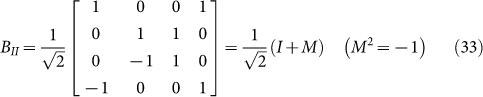
and
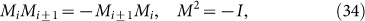

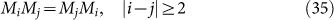
which forms “extra special 2-group”. Obviously, M is extension of i for i^2^ = −1.

An interesting observation is^28^:
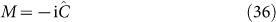
where 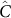 is the charge conjugate operator in Majorana spinor. The eigenstates of 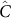 take the forms
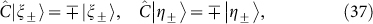
where
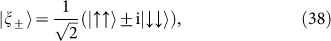

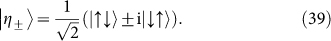


Here we would like to give an intuitive interpretation of the operator Γ in Ref. 8 by taking a new set of *D_i_* (*i* = 1, 2, 3) in stead of *γ_i_*, and show how it gives rise to the Majorona doubling with explicit realization.

We follow the concrete realization for *γ_j_* given in Ref. 8, (in this paper *I* is 2 × 2 identity matrix)
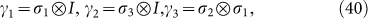

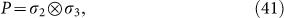

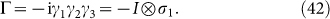


In our notation, 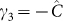, i.e. (38) and (39) are eigenstates of *γ*_3_. It is easy to find
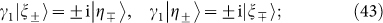

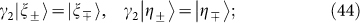

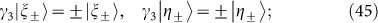

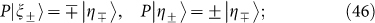

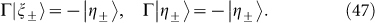


In the derivation of (43)–(47), the relations *σ*_1_ = (*S*^+^ + *S*^−^) and 
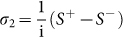
 have been used where *S*^±^ = *S*_1_ ± i*S*_2_. To show the importance of Γ-operator we define new Clifford algebra {*D_i_*, *D_j_*} = 2*δ_ij_*, where *D*_1_ = *γ*_2_, *D*_2_ = Γ*γ*_1_, *D*_3_ = *γ*_3_. It is interesting to find that
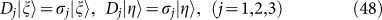

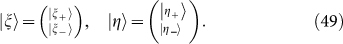


Namely, by acting *D_j_* on |*ξ*〉 or |*η*〉, the representation is exactly Pauli matrices, i.e. belonging to SU(2) algebra. It can be checked that
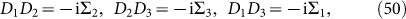
where Σ*_i_* form the reducible representation of *SU* (2):
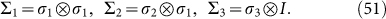


The introduced interacting Hamiltonian *H_B_* = −i(*αD*_1_*D*_2_ + *βD*_2_*D*_3_ + *κD*_3_*D*_1_) can be recast to
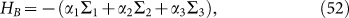
where *α*_1_ = −*κ*, *α*_2_ = *α*, *α*_3_ = *β*. Noting that *D*_1_*D*_2_*D*_3_ = −i*I* ⊗ *I*, i.e. trivial. The direct check gives:
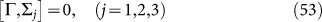
and
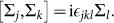
Then the *H_B_* can be written in the form:
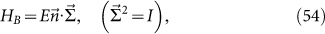

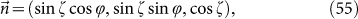

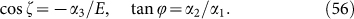
Obviously, 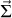 is reducible 4-d representation of SU(2). Explicitly,
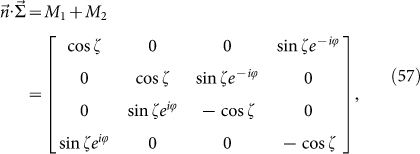
where
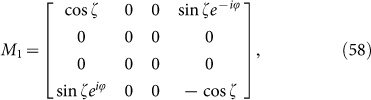

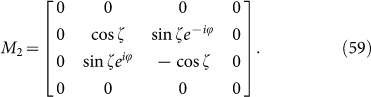
Rewriting *M*_1_ and *M*_2_ in the form of Pauli matrices, we have
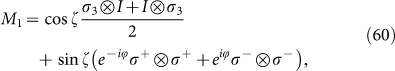

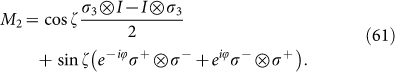


Now the meaning of *H_B_* is manifest: 4-dimension is quite different from 2-dimension. The “edge block” leads to *M*_1_ with superconducting type of Hamiltonian whereas “interior block” *M*_2_ is connected with the usual spin chain. It is easy to find the eigenstates of *M*_1_ and *M*_2_:
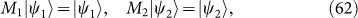
where
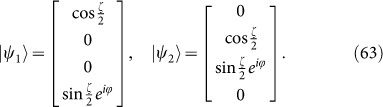


Acting Γ on (63) it yields
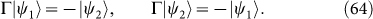
So Γ transforms between |*ψ*_1_〉 and |*ψ*_2_〉 that holds for the same energy. It never occurs in 2 dimensions. Meanwhile, equation (53) shows that Γ commutes with the Hamiltonian *H_B_*, which means that Γ-transformation does not change the property of Hamiltonian *H_B_*. This example shows that operator Γ is crucial in leading to Majorana doubling in dimensions ≥4. With the new defination of *D*_2_, we should define a new parity operator:
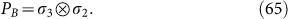
Direct check gives the complete set of algebra
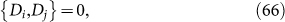

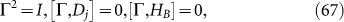

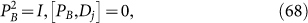

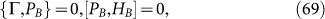

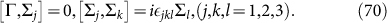


It is noteworthy that the introduced *P_B_* in equation (68) commutes with *D_j_* instead of the anti commuting relation between *P* and *γ_j_*. And *P_B_* still anticommutes with Γ. Acting *P_B_* on the eigenstates |*ψ*_1_〉 and |*ψ*_2_〉, it follows
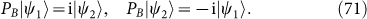


In such a concrete realization Γ plays the essential role. The Hamiltonian (54) formed by (52) looks a typical nuclear resonant model in 4 dimensions. Only the higher dimensions allow the operator Γ leading to the doubling degeneracy.

### Majorana doubling in 3-body Hamiltonian based on YBE

Now we discuss the interaction of 3 Majorana fermions based on YBE.

It is well known that 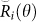 describes the 2-body interaction. And the physical meaning of Yang-Baxter equation is that the interaction of the three bodies can be decomposed into three 2-body interactions:
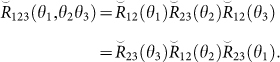
Because of the constraint in equation (11), 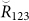 depends only on two free parameters and has the following form^29^:
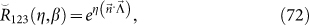
where
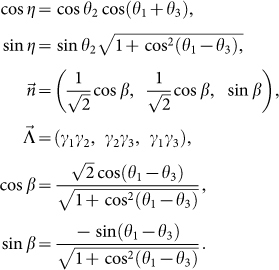
Here the parameters *θ*_1_ and *θ*_3_ are replaced by *η* and *β*. 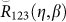 is also a unitary operator and describes the interaction of three Majorana operators.

We suppose that the parameter *η* is time-dependent and *β* is time-independent in 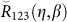, then the desired 3-body Hamiltonian can be obtained from equation (22):
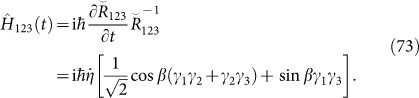
The constructed Hamiltonian, which has been mentioned in Ref. 7, 8, describes the 2-body interactions among the three Majorana operators. It describes the effective interaction in a *T*-junction formed by three quantum wires. In Ref. 8, it has been shown that the above Hamiltonian, which commutes with emergent Majorana operator Γ = −iγ_1_γ_2_γ_3_, holds Majorana doubling. From the viewpoint of YBE, the intrinsic commutation relation is between Γ and the solution of YBE 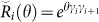. It is shown that:
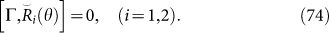
Indeed, the above commutation relation indicates that emergent Majorana operator Γ is a new symmetry of the solution 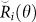 of YBE. It is due to the decomposition of 3-body interaction into three 2-body interactions via the approach of YBE that the derived Hamiltonian holds Majorana doubling.

The extended emergent Majorana mode Γ′ supporting odd number *N* of Majorana operators^8^ is,
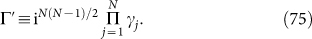
It is easy to check that:
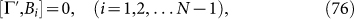
where 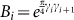 is the generator of the braid group *B_N_*. The commutation relation indicates that Γ′ plays the role of an invariant in the braid group *B_N_*.

### Generation of 2n-qubit GHZ state via YBE

Quantum entanglement plays an important role in quantum information theory and has been discussed in both theoretical^30^ and experimental^31^,^32^,^33^ aspects for a long time. There are various ways in describing different types of entanglement. It is also well known that the relationship between Yang-Baxter equation and 2-qubit entangled state as well as 3-qubit entanglement has been discussed in Ref. 22, 23, 29, 34. Here we construct high dimensional matrix representation of solution to Yang-Baxter equation and discuss how it generates 2*N*-qubit GHZ state for odd *N*. In previous section, we present Clifford algebric relation for different Majorana operators,
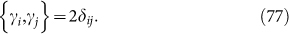
It can be used for constructing solution to YBE:
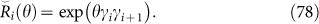
The representation of *γ_i_* in the Majorana form is given by:
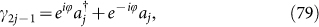

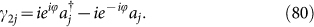
Then by constructing Yang-Baxter chain, we find its similarity to 1D Kitaev model.

Indeed, the 4D-matrix representation is equivalent to the Majorana fermion representation under Jordan-Wigner transformation. In other words, we can express *γ_i_* by matrix directly. For three operators. *γ*_1_, *γ*_2_ and *γ*_3_ satisfying Clifford algebra, its 4D matrix representation has been presented in Ref. 8:
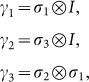
here *σ_i_* are Pauli matrices.

What we are interested in is constructing higher dimensional matrix representation of *γ_i_*. Taking 8D representation as an example, *γ_i_* is:
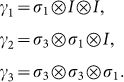
Then the matrix form of emergent Majorana mode Γ^8^ is,
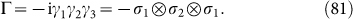
The Hamiltonian supporting three Majorana operators has been defined in equation (2):
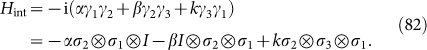
Obviously, Γ commutes with the Hamiltonian *H*_int_.

Let us extend Γ to N sites Γ*_i_*, which should also satisfy Clifford algebra {Γ*_i_*, Γ*_j_*} = 2*δ_ij_*. The Γ*_i_* has the following form:
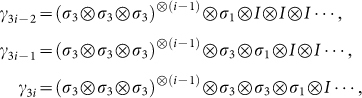

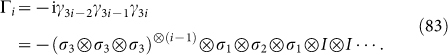
Then we have:
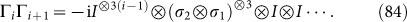
It is easy to check that 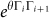 is the 4^3^-D matrix solution of YBE, we denote it by 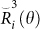,
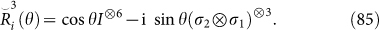
By acting 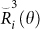 on 6-qubit natural basis, such as |↑↑↑↑↑↑〉, we have:
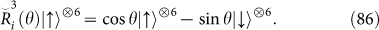
This state represents a type of 6-qubit entangled states. In the case of 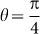, the generated state is 6-qubit GHZ state, and 
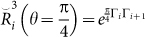
 can be regarded as one braiding operation of two emergent Majorana operator Γ*_i_* and Γ*_i_*_ + 1_.

Now we generalize the 4^3^−D matrix solution of YBE to 4*^n^* with *n* odd. The extended Majorana operator supporting any odd number *n* of Majorana operators reads,
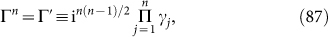
where the constraint of Clifford algebra 
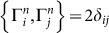
 leads to the odd number *n*. 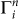 can be expressed as:
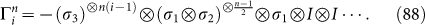
Then we have
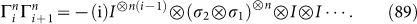
The 4*^n^*−D (*n* odd) matrix representation of solution to YBE is:
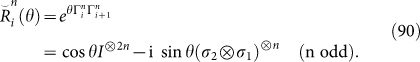
Consequently, we generate the following state by acting 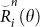 on the 2*n*(*n* odd)-qubit natural state |↑〉^⊗2*n*^:
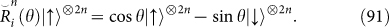
When 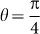, the generated state turns into 2*n*-qubit GHZ state for odd *n*.

## Discussion

In this paper, based on the solution of YBE in Majorana form, we discuss the topological phase transition in the derived “superconducting” chain and the Majorana doubling in 3-body Hamiltonian as well as the generation of 2n-qubit GHZ-type entangled states. Unlike the braid operator, the solution 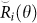 of YBE is parameter-dependent. Hence the unitary operator 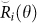 can be used for generating the “superconducting” chain and the Majorana doubling in 3-body Hamiltonian. Indeed, the derived chain(25,26) describes the braiding transformation of nearest-neighbour Majorana sites for 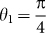 (or 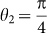). We also find that the 3-body Hamiltonian 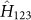 derived from 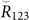 holds Majorana doubling. From the viewpoint of YBE, the commutation relation 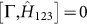 can be explained by 
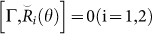
, where 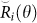 is the solution of YBE. In other words, it is the Γ-symmetry of 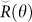 that leads to the Γ-symmetry of 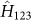. The commutation relation can also be generalized to the extended Γ′-operator(87) for odd *N* sites, [Γ′, *B_i_*] = 0 (*i* = 1, 2, …*N* − 1), hence Γ′ is an invariant of the braid group *B_N_*.

We present a new realization of Majorana doubling based on emergent Majorana mode and show the role of Γ in leading to the doubling degeneracy of *H_B_* intuitively. We also make use of the extended Γ′-operator to construct high dimensional matrix representation of solution to YBE. By acting the high dimensional matrix representation of solution of YBE on natural basis, we generate the GHZ-type entangled state. Thus we conclude that the braiding process of the extended Γ′-operators corresponds to the generation of GHZ entangled state. These results may guide us to find much closer relationship between Yang-Baxter equation and quantum information as well as condensed matter physics.

## Author Contributions

M.L.G. proposed the idea, L.W.Y. performed the calculation and derivation, L.W.Y. and M.L.G. prepared the manuscript, all authors reviewed the manuscript.
